# Restoring Angiotensin Type 2 Receptor Function Reverses PFOS-Induced Vascular Hyper-Reactivity and Hypertension in Pregnancy

**DOI:** 10.3390/ijms241814180

**Published:** 2023-09-16

**Authors:** Sri Vidya Dangudubiyyam, Bradley Bosse, Pankaj Yadav, Ruolin Song, Alissa Hofmann, Jay S. Mishra, Sathish Kumar

**Affiliations:** 1 Department of Comparative Biosciences, School of Veterinary Medicine, University of Wisconsin, Madison, WI 53706, USA; sdangudubiyy@wisc.edu (S.V.D.); pyadav23@wisc.edu (P.Y.); ruolin.song@wisc.edu (R.S.); ahofmann2@wisc.edu (A.H.); jay.mishra@wisc.edu (J.S.M.); 2 Endocrinology-Reproductive Physiology Program, University of Wisconsin, Madison, WI 53715, USA; 3 Department of Obstetrics and Gynecology, School of Medicine and Public Health, University of Wisconsin, Madison, WI 53792, USA; bbosse@wisc.edu

**Keywords:** PFOS, AT2R agonist, blood pressure, uterine blood flow, angiotensin II, endothelium, vascular function

## Abstract

Perfluorooctane sulfonic acid (PFOS) exposure during pregnancy induces hypertension with decreased vasodilatory angiotensin type-2 receptor (AT2R) expression and impaired vascular reactivity and fetal weights. We hypothesized that AT2R activation restores the AT1R/AT2R balance and reverses gestational hypertension by improving vascular mechanisms. Pregnant Sprague-Dawley rats were exposed to PFOS through drinking water (50 μg/mL) from gestation day (GD) 4–20. Controls received drinking water with no detectable PFOS. Control and PFOS-exposed rats were treated with AT2R agonist Compound 21 (C21; 0.3 mg/kg/day, SC) from GD 15–20. In PFOS dams, blood pressure was higher, blood flow in the uterine artery was reduced, and C21 reversed these to control levels. C21 mitigated the heightened contraction response to Ang II and enhanced endothelium-dependent vasorelaxation in uterine arteries of PFOS dams. The observed vascular effects of C21 were correlated with reduced AT1R levels and increased AT2R and eNOS protein levels. C21 also increased plasma bradykinin production in PFOS dams and attenuated the fetoplacental growth restriction. These data suggest that C21 improves the PFOS-induced maternal vascular dysfunction and blood flow to the fetoplacental unit, providing preclinical evidence to support that AT2R activation may be an important target for preventing or treating PFOS-induced adverse maternal and fetal outcomes.

## 1. Introduction

Hypertensive disorders in pregnancy (HDPs) encompass pre-pregnancy (chronic) or pregnancy-associated hypertension and represent prevalent pregnancy complications in the United States. The incidence of HDPs has been on the rise, affecting approximately 15% of women during their reproductive years [1]. HDPs are significantly linked to severe maternal complications, including heart attack and stroke [2], and remain a primary cause of pregnancy-related mortality in the United States [1]. Moreover, mothers who survive HDPs and their offspring face an elevated risk of enduring long-term health complications, including the development of cardiovascular and metabolic diseases [3,4,5,6,7,8,9,10]. Despite the severe threat HDPs pose to both maternal and fetal health, the underlying mechanism remains unclear. It is proposed that endothelial dysfunction, resulting in inadequate hemodynamic alterations, plays a role in the pathogenesis of HDPs [11]. Due to this uncertain pathogenesis, the available treatment options for HDPs are presently limited.

Known risk factors for HDPs include advanced age at first pregnancy and increasing prevalence of obesity and other cardiometabolic risk factors [2,12]. In recent years, exposure to environmental pollutants, such as perfluorooctane sulfonate (PFOS) during pregnancy, has been linked to unfavorable maternal outcomes, including gestational hypertension, preeclampsia, and fetal growth restriction [13,14,15,16,17,18,19,20]. PFOS is a member of the perfluoralkyl substances (PFAS) family, comprising approximately 5000 synthetic compounds widely utilized in diverse commercial products and manufacturing processes due to their resistance to extreme temperatures, degradation, and nonstick properties [21,22]. Owing to their extensive usage and stability, these chemicals have become pervasive in the environment and human populations [23]. Despite efforts to phase out PFOS production and reduce exposure, PFOS continues to be detected in most water sources in the US and globally [24,25]. PFOS exposure in humans primarily occurs through drinking water and diet, but it can also arise from sources like house dust, air, cleaning products, and consumer goods [26]. The human half-life of PFOS ranges from 3.3 to 6.9 years [27,28]. PFOS is minimally metabolized [29], poorly eliminated [30,31], and exhibits bioaccumulation in blood and various tissues [32].

PFOS is a reproductive toxicant and also adversely affects cardiovascular function [33,34,35]. The health-related costs of PFAS exposure were estimated to be $37–59 billion annually in the United States [36]. Studies indicate that PFOS induces vascular injury with significant reductions in the formation and length of intersegmental and posterior cardinal veins while enhancing dorsal aorta vessel formation in hatched zebrafish embryos [37]. Furthermore, PFOS exerts proinflammatory effects on human umbilical vein endothelial cells with alterations in actin filament remodeling, increased generation of reactive oxygen species, and disruptions in adhesion junction integrity and endothelial permeability barriers [38,39,40]. PFOS was shown to decrease vessel formation in a 3D model of human umbilical vein endothelial cells and colon fibroblast co-culture [41] and impedes cell growth, migration, and angiogenesis in human HTR-8/SVneo and JEG-3 cells [42]. Recent studies in pregnant rats demonstrated that PFOS exposure negatively impacts endothelial cell function by reducing endothelial nitric oxide synthase (eNOS) expression and impairing endothelium-dependent vascular relaxation [43]. Additionally, PFOS induces gestational hypertension by causing hypersensitivity and exaggerated vascular contractile responses to angiotensin II (Ang II) [43]. These findings collectively indicate that PFOS affects vascular and endothelial function both in vivo and in vitro. Identifying a specific PFOS-affected mechanism holds promise for developing preventative and therapeutic strategies.

The renin-angiotensin system (RAS) plays a crucial role in regulating blood pressure and blood flow to the uteroplacental unit during gestation [44,45]. Angiotensin II (Ang II), the primary effector of the RAS, exerts its effects through two main receptors, namely AT1R and AT2R. AT1R mediates vasoconstriction and hypertensive effects, while AT2R promotes vasodilation and enhances blood flow [46]. Clinical and experimental research has indicated that increased AT1R levels and decreased AT2R protein levels are associated with the development of preeclampsia [47,48]. Interestingly, restoring AT2R expression and function in both preeclampsia patients and animal models has been shown to prevent pathological outcomes [47]. Recently, our study demonstrated that exposure to PFOS during pregnancy leads to gestational vascular dysfunction, characterized by reduced AT2R protein expression and increased AT1R levels [43]. Although the dysregulation of AT1R and AT2R expression and function could contribute to gestational hypertension and preeclampsia, the specific role of AT2R in regulating vascular function in the setting of exposure to environmental pollutants like PFOS remains unclear. Also, whether AT2R activation could correct the imbalance in AT1R/AT2R expression in PFOS-exposed dams is unclear. The present study was designed to test the hypothesis that AT2R activation with Compound 21 (C21) restores the AT1R/AT2R balance and reverses gestational hypertension by improving vascular function and vascular contraction and relaxation mechanisms.

## 2. Results

### 2.1. Blood Pressure and Uterine Artery Blood Flow in Pregnant Rats

**Figure 1:**
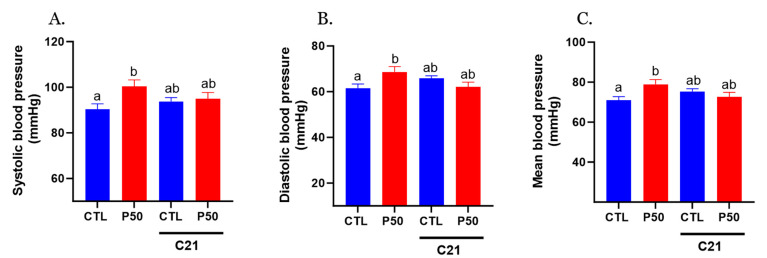
Effect of AT2R agonist C21 treatment on maternal blood pressure. Pregnant rats were exposed to PFOS via drinking water (50 μg/mL) from gestation day 4 to 20. Controls received PFOS-free drinking water. Both control and PFOS-exposed groups were treated with AT2R agonist C21 from GD 15 to 20. On GD 20, (**A**) systolic, (**B**) diastolic, and (**C**) mean arterial blood pressure were measured noninvasively using the CODA system. Data are presented as means ± SEM of 6 rats per group. Means with different letters indicate significant differences (*p* ≤ 0.05) among the groups.

PFOS dams exhibited elevated systolic, diastolic, and mean blood pressure, and administration of AT2R agonist C21 prevented PFOS-induced increase in blood pressure (Figure 1; *p* ≤ 0.05; n = 6). However, C21 did not significantly impact blood pressure in the control group (Figure 1; *p* ≤ 0.05; n = 6).

**Figure 2:**
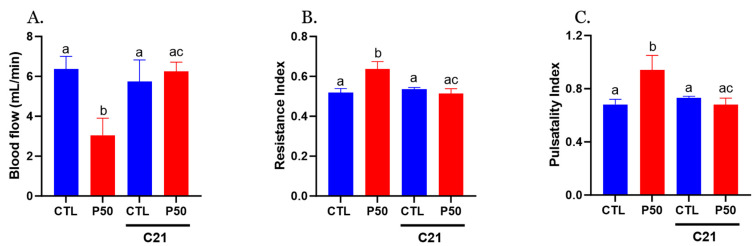
Effect of AT2R agonist C21 treatment on uterine artery hemodynamics. (**A**) Uterine artery blood flow, (**B**) resistance index, and (**C**) pulsatility index were measured using a 30-MHz transducer and Vevo 2100 micro-ultrasound on GD 20 in Control and PFOS dams with and without C21. Data are expressed as means ± SEM of 6 rats per group. Means with different letters indicate significant differences (*p* ≤ 0.05) among the groups.

Furthermore, PFOS dams demonstrated a significant reduction in uterine artery blood flow, accompanied by increased resistance and pulsatility indices compared to the control group (Figure 2; *p* ≤ 0.05; n = 6). Administration of C21 significantly restored uterine artery blood flow and normalized resistance and pulsatility indices to control levels. No significant uterine artery hemodynamic effects were observed with C21 treatment in the control group (Figure 2; *p* ≤ 0.05; n = 6).

### 2.2. Vasoconstrictor Response

**Figure 3:**
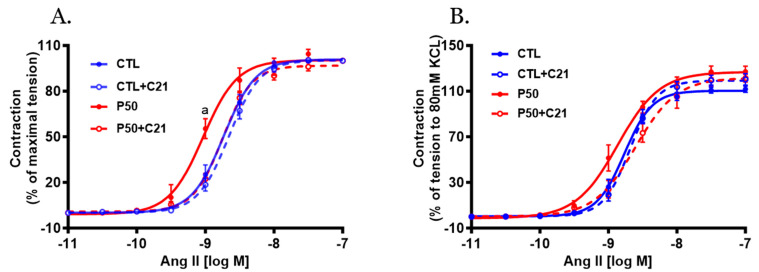
Effect of AT2R agonist C21 treatment on Angiotensin II (Ang II)-mediated uterine artery contractile responses. On gestation day 20, uterine artery rings were isolated from pregnant rats exposed to control conditions and PFOS, both with and without C21 treatment. Vascular contractile responses to cumulative Ang II additions were measured and presented as (**A**) percentage of maximal contraction and (**B**) percentage of contraction induced by 80 mM KCl. The data represent means ± SEM of 6 rats per group. ^a^ *p* ≤ 0.05 compared to all other groups.

PFOS dams exhibited greater Ang II-induced contractile responses in endothelium-denuded uterine arteries, characterized by increased sensitivity compared to controls (Figure 3 and Table 1; *p* ≤ 0.05; n = 6). However, administration of C21 significantly attenuated the PFOS-induced exaggerated Ang II contraction (Figure 3 and Table 1; *p* ≤ 0.05; n = 6). C21 did not elicit any significant effects on Ang II vasoconstriction in controls (Figure 3 and Table 1; n = 6).

**Figure 4:**
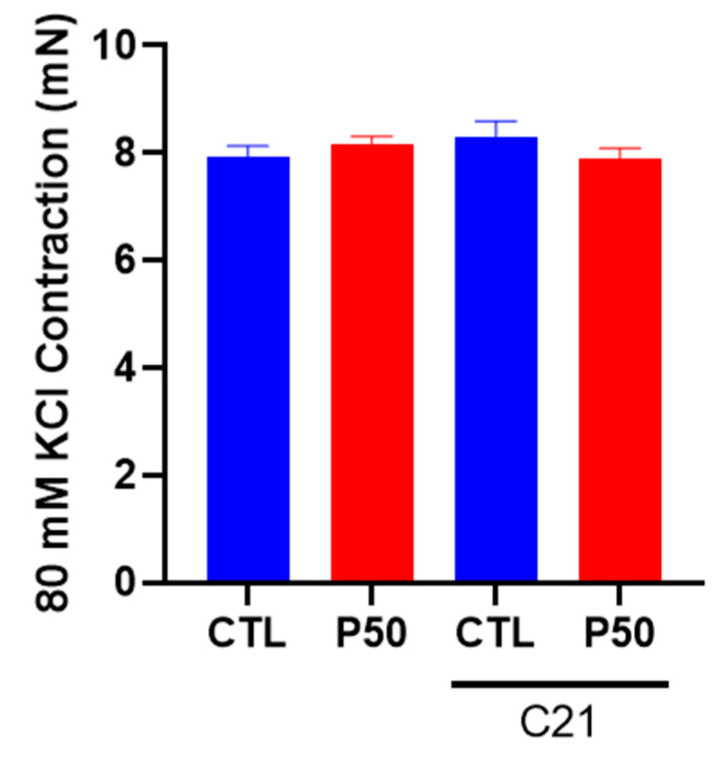
Effect of AT2R agonist C21 treatment on depolarization-induced uterine artery contractile responses to potassium chloride (KCl). On gestation day 20, contractile responses to 80 mM KCl were assessed in endothelium-denuded uterine arteries from pregnant rats exposed to control conditions and PFOS, both with and without C21 treatment. The data are presented as means ± SEM of 6 rats per group.

The vascular contractile responses to KCl (80 mM), a determinant of depolarization-induced contraction, were similar in PFOS and control dams (Figure 4; n = 6). C21 treatment did not induce significant alterations in the KCl-induced contraction in either PFOS or control dams (Figure 4; n = 6).

### 2.3. Vasodilator Response

**Figure 5:**
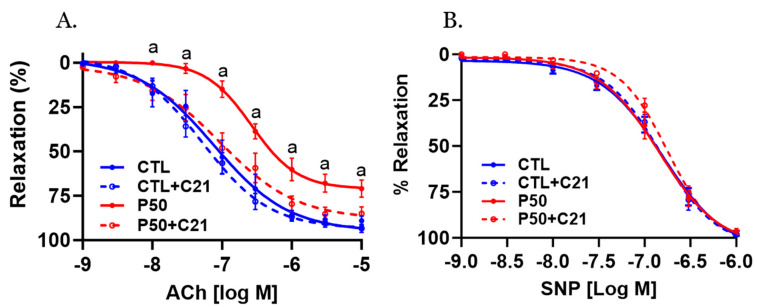
Effect of AT2R agonist C21 treatment on endothelium-dependent vascular relaxation responses. On gestation day 20, uterine artery rings from pregnant rats exposed to control conditions and PFOS, both with and without C21 treatment, were pre-contracted using submaximal phenylephrine. Subsequently, the relaxation responses to cumulative concentrations of (**A**) acetylcholine (ACh) and (**B**) sodium nitroprusside (SNP) were examined. The data are presented as means ± SEM of 6 rats per group. ^a^ *p* ≤ 0.05 compared to all other groups.

Acetylcholine (ACh)-induced relaxation was significantly reduced in endothelium-intact uterine arteries with decreased ACh sensitivity and maximal response in PFOS dams compared to controls (Figure 5A and Table 1; *p* ≤ 0.05; n = 6). However, C21 restored the decreased ACh relaxation in PFOS dams by increasing ACh sensitivity and maximal relaxation (Figure 5A and Table 1; *p* ≤ 0.05; n = 6). No significant changes in ACh-induced relaxation responses were observed in the control group following C21 treatment (Figure 5A and Table 1; n = 6).

The nitric oxide (NO) donor Sodium nitroprusside (SNP) induced concentration-dependent relaxation that was equally potent in control and PFOS dams, with and without C21 treatment (Figure 5B and Table 1; n = 6).

### 2.4. Ang II receptors and eNOS Protein Levels

**Figure 6:**
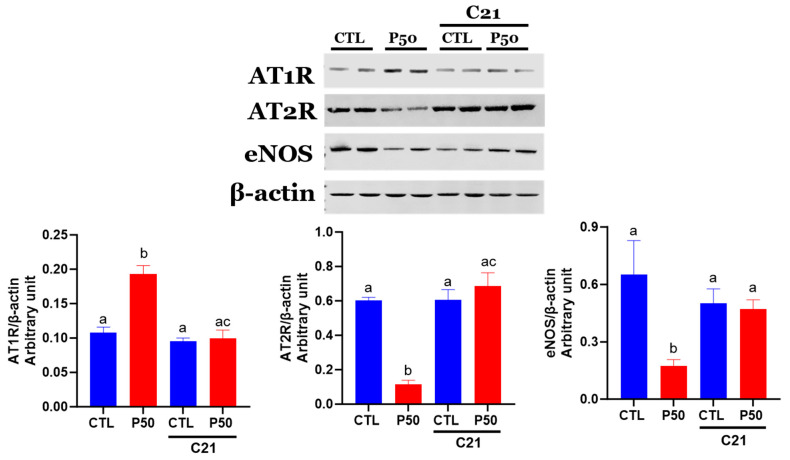
Effect of AT2R agonist C21 treatment on protein expression of Ang II receptors and eNOS in the uterine arteries. Protein expression of AT1R, AT2R, and eNOS in GD 20 uterine artery samples from pregnant rats exposed to control conditions and PFOS, both with and without C21 treatment, were analyzed using Western blotting. The left panel displays representative blots for AT1R, AT2R, eNOS, and β-actin, while the right panel shows normalized densitometry data. Data are expressed as means ± SEM of 6 rats per group. Means with different letters indicate significant differences (*p* ≤ 0.05) among the groups.

As shown in Figure 6, uterine arteries from PFOS dams exhibited increased AT1R protein levels, while AT2R and eNOS protein levels were reduced compared to controls (Figure 6; *p* ≤ 0.05; n = 6). In contrast, C21 administration to PFOS dams decreased AT1R protein levels and increased AT2R and eNOS protein levels, but it did not significantly affect the control group (Figure 6; *p* ≤ 0.05; n = 6).

### 2.5. Plasma Bradykinin Levels

**Figure 7:**
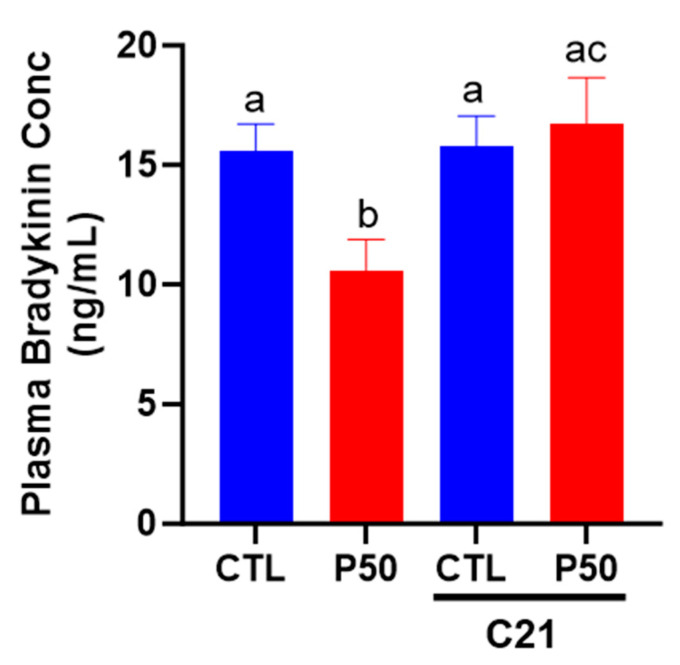
Effect of AT_2_R agonist C21 on plasma bradykinin levels. On gestation day 20, blood was collected from pregnant rats exposed to control conditions and PFOS, both with and without C21 treatment, via cardiac puncture following CO_2_ inhalation. Bradykinin levels were assessed using an ELISA kit. The data are presented as means ± SEM of 6 rats per group. Means with different letters indicate significant differences (*p* ≤ 0.05) among the groups.

As shown in Figure 7, PFOS dams had a significant reduction in plasma bradykinin levels compared to controls (*p* ≤ 0.05; n = 6). Conversely, C21 administration ameliorated the PFOS-induced decline in bradykinin levels. The plasma levels of bradykinin remained unaffected in control dams with C21 treatment (Figure 7; *p* ≤ 0.05; n = 6).

### 2.6. Placental and Fetal Weight

**Figure 8:**
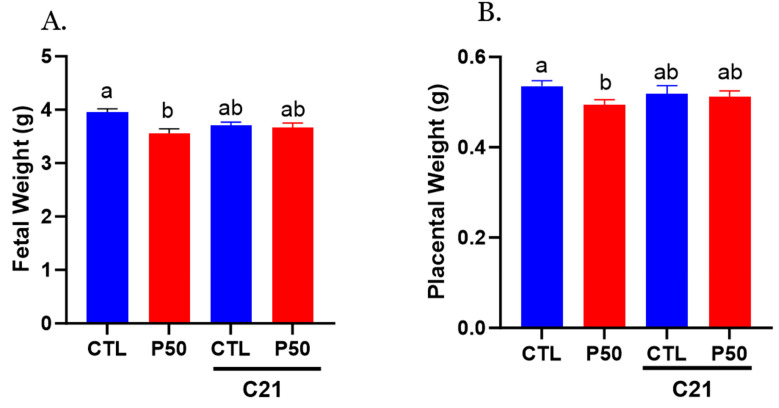
Effect of AT_2_R agonist C21 on fetal and placental weights. On gestation day 20, fetal and placental weights were measured in pregnant rats exposed to control conditions and PFOS, both with and without C21 treatment. (**A**) Fetal weights and (**B**) placental weights were averaged for each dam, and each dot represents the mean data per dam/litter. The data are presented as means ± SEM of 6 rats per group. Means with different letters indicate significant differences (*p* ≤ 0.05) among the groups.

As shown in Figure 8A,B, elevated maternal PFOS resulted in placental and fetal growth restriction. However, treatment with C21 significantly mitigated the adverse effects of PFOS by rescuing the placental and fetal weight (Figure 8A,B; *p* ≤ 0.05; n = 6). C21 administration did not significantly impact the placental and fetal weights of control dams (Figure 8A,B; *p* ≤ 0.05; n = 6). Furthermore, C21 treatment did not induce any alteration in the litter size of control and PFOS dams (Table 2).

## 3. Discussion

The major findings of the current study are as follows: (1) Administration of the AT2R agonist C21 to PFOS-exposed dams effectively mitigated PFOS-induced hypertension in pregnant rats by improving uterine artery blood flow and attenuating Ang II-mediated vascular contraction. Furthermore, C21 administration enhanced endothelial-dependent vascular relaxation in PFOS-exposed dams. (2) The suppression of Ang II-mediated vascular contraction by C21 in PFOS-exposed dams was correlated with decreased AT1R receptors and increased AT2R receptors within the uterine artery. (3) The enhanced endothelial-dependent relaxation response observed in C21-treated PFOS dams was associated with elevated eNOS expression in the uterine artery with increased plasma bradykinin levels. (4) C21 treatment also improved feto-placental growth in PFOS-exposed dams, likely attributed to improved vasodilation and enhanced uterine artery blood flow. These findings represent a significant and novel contribution, suggesting that activation of the AT2R receptor by C21 attenuates PFOS-induced hypertension, enhances endothelial-mediated vascular function, and improves feto-placental growth in pregnant rats exposed to PFOS.

Emerging evidence substantiates a link between maternal exposure to PFOS and various detrimental maternal outcomes, including gestational hypertension, preeclampsia [13,14,15,16,17,18,19,20], and decreased birth weight [49,50,51,52,53,54,55,56,57]. Furthermore, our recent study revealed that PFOS exposure dose-dependently increased mean arterial pressure in pregnant Sprague Dawley rats [43]. In the present study, we observed elevated blood pressure in pregnant rats exposed to PFOS, consistent with previous studies [43,58]. However, the administration of the AT2R agonist C21 to PFOS-exposed dams effectively prevented the blood pressure increase, suggesting that C21 may have the ability to attenuate PFOS-induced hypertension. This finding aligns with earlier studies wherein C21 demonstrated the restoration of blood pressure in various hypertensive models, including testosterone-induced hypertension in pregnant Sprague Dawley rats [47], Ang II-induced hypertension in non-pregnant female Sprague Dawley rats [59], and salt-induced hypertension in male obese Zucker rats [60]. Nevertheless, our study represents the first to demonstrate the potential of AT2R activation by C21 in alleviating hypertension caused by environmental chemical exposure, such as PFOS.

During pregnancy, maternal vascular adaptations are crucial in increasing uterine artery blood flow to meet the metabolic demands of the developing placenta and fetus [61,62]. In the present study, exposure to PFOS decreased uterine artery blood flow and increased resistance and pulsatility indices on GD20, which aligns with our recent investigation [43]. Reduced uterine artery blood flow and increased vascular resistance have been associated with adverse outcomes such as preeclampsia and fetal growth restriction [63,64]. In our study, administration of C21 to PFOS-exposed dams improved uterine artery blood flow and restored the resistance and pulsatility indices. The potential of C21 to enhance blood flow has also been observed in other studies, wherein C21 increased uterine blood flow in hyperandrogenic pregnant rats [47] and enhanced renal blood flow in male and female ten-week-old Sprague Dawley rats [65]. Uterine vascular remodeling and placental angiogenesis are critical processes involved in establishing a “low resistance, high capacitance vessel” capable of augmenting uterine blood flow [66,67]. Notably, significant changes occur in uterine spiral and placental arteries, including increased branching, diameter, and total area, which contribute to enhanced uterine blood flow [68]. Exposure to PFOS is reported to inhibit angiogenesis in the placenta [69] and in human umbilical vein endothelial cells [41] and reduce placental vascular density [70]. In contrast, C21 has been shown to induce angiogenesis and upregulate multiple angiogenic proteins [71]. Therefore, it is plausible to suggest that C21 treatment in PFOS-exposed dams may improve angiogenesis and enhance placental vascularization, ultimately increasing uterine artery blood flow. However, further investigations are necessary to elucidate the specific role of C21 in vascular remodeling during PFOS exposure.

We investigated uterine artery function to elucidate the potential vascular mechanisms underlying the observed decrease in blood pressure and increase in uterine artery blood flow associated with C21 treatment in PFOS-exposed dams. The increased blood pressure observed in PFOS dams was accompanied by enhanced vasoconstriction in response to Ang II, resembling the Ang II hyperreactivity observed in hypertensive pregnancies [43]. However, administration of C21 to PFOS dams in our study effectively reversed the exaggerated vasoconstriction in response to Ang II, restoring it to levels comparable to those observed in Control pregnant rats supporting the involvement of AT2R activation in restoring vascular function in PFOS-exposed dams. Importantly, the response to KCl, which induces contraction through membrane depolarization, did not differ significantly between endothelium-denuded uterine arteries with and without C21 treatment. This suggests that the attenuated Ang II vasoconstriction observed in PFOS dams treated with C21 is more likely attributed to changes in Ang II receptors rather than generalized nonreceptor-mediated alterations, such as hypertrophy or hyperplasia of vascular smooth muscle cells. Supporting this notion, uterine arteries from PFOS dams exhibited increased protein abundance of AT1R and decreased protein abundance of AT2R, while C21 treatment successfully restored the balance of angiotensin receptors in the uterine arteries of PFOS-exposed dams. The exact mechanism by which AT2R activation decreases AT1R abundance is still not fully understood, but several animal studies suggest the involvement of a complex cross-regulatory mechanism between AT1R and AT2R [47,72,73,74]. For instance, in both in vitro and in vivo investigations, it has been observed that AT2R stimulation has the capacity to influence AT1R expression, suggesting the existence of intricate cross-regulatory mechanisms between these receptors. Specifically, AT2R stimulation or overexpression has been found to inhibit AT1R expression and signaling [73,74]. In AT2R knockout mice, AT1R expression was elevated in vascular tissues compared to control mice [74]. Moreover, introducing the AT2R gene into rat vascular smooth muscle cells resulted in the inhibition of AT1R-mediated tyrosine phosphorylation of signal transducers and activators (STAT) [73]. Conversely, blocking AT1R led to an upregulation of AT2R expression in vascular smooth muscle cells, suggesting a reciprocal regulation by AT1R [75]. Furthermore, in endothelial cells transfected with the AT2R promoter, AT1R stimulation attenuated AT2R expression [76]. Therefore, the attenuation of exaggerated Ang II vasoconstriction in PFOS dams treated with C21 may be due to the decreased abundance of vasoconstrictive AT1R receptors in uterine arteries of C21-treated PFOS dams.

In order to investigate the effects of C21 on endothelial function, we assessed the endothelium-dependent relaxation response to ACh. Notably, ACh-induced relaxation was reduced in the uterine arteries of PFOS dams, indicating impaired endothelial Control of vascular tone, which is consistent with a previous study [43]. Intriguingly, C21 treatment enhanced ACh-induced relaxation in the uterine arteries of PFOS dams while exerting minimal effects in control dams. These findings suggest that the vascular relaxation responses to ACh are preserved in the presence of C21 treatment. Importantly, the relaxation response to the NO donor SNP did not differ significantly between the C21-treated PFOS dams and PFOS dams, indicating that the observed differences were not related to the vasodilatory capacity of smooth muscle but rather to endothelial function. Consistently, previous studies have demonstrated that C21 improves endothelium-dependent NO-mediated relaxations in spontaneously hypertensive and hyperandrogenic rats [47,77]. These results suggest that AT2R activation may enhance NO synthesis by endothelial cells. This concept is supported by the observation that the levels of eNOS protein were increased in the uterine arteries of C21-treated PFOS dams. Although the exact mechanism by which C21 triggers eNOS expression is not fully understood, previous reports have suggested that C21 can directly stimulate eNOS expression in placental arteries [47]. Moreover, in a mouse model of diet-induced obesity, C21 preserved eNOS levels through PKA/p-eNOS and AKT/p-eNOS signaling pathways [78]. It was interesting to note that PFOS exposure decreased plasma bradykinin levels. The precise mechanism by which PFOS reduces bradykinin levels remains unclear. However, activation of AT2R increased bradykinin production, which is consistent with previous reports [47,78]. As bradykinin is known to induce vasodilation through increased production of NO, prostacyclin, and endothelium-derived hyperpolarizing factors, the improvement in vasodilation and uterine artery blood flow observed in the C21-treated PFOS dams may also be attributed to the increase in bradykinin levels [79,80,81]. Therefore, the present study provides evidence supporting the role of AT2R activation in preserving endothelium-dependent vasodilation in PFOS-exposed dams.

The association between PFOS exposure and adverse outcomes, including fetal growth restriction and low birth weight, has been consistently observed in both human [49,50,51,52,53,54,55,56,57] and animal studies [58,82,83,84,85,86,87,88,89]. In our present study, treatment with C21 significantly improved the weights of both the placenta and fetus in PFOS-exposed dams. These findings suggest that the beneficial effect of C21 treatment in PFOS dams may be attributed, at least in part, to the improvement in vascular function and enhanced uterine artery blood flow. Studies show that PFOS exposure decreases transplacental glucose and amino acid transport to the fetus [90]. It would be interesting to examine if AT2R activation improves nutrient availability in the fetus.

In summary, this study concurs with the previous report that PFOS exposure during pregnancy disrupts endothelial function, resulting in hypertension in rats by suppressing AT2R-mediated vasodilation [43]. This study provides new information that activation of AT2R using a pharmacological agonist (C21) in PFOS-exposed dams restores the balance of Ang II receptors, leading to optimal blood pressure, enhanced uterine artery blood flow, reduced Ang II vasoconstriction, improved endothelial-mediated relaxation, and enhanced feto-placental growth. It is important to note that the present findings specifically highlight the mitigatory effect of C21 on vascular hemodynamics in PFOS-exposed dams, and caution should be exercised in generalizing these results to other PFOS-induced adverse outcomes. Future investigations should explore the potential of C21 or other AT2R agonists in reversing additional PFOS-related complications. Nonetheless, these results suggest that augmenting AT2R activity through pharmacological agonists holds promise as a preventive or therapeutic strategy for managing gestational hypertension and fetal growth restriction associated with PFOS exposure.

## 4. Materials and Methods

### 4.1. Animals

All animal procedures were conducted in accordance with the guidelines set by the US National Institutes of Health (NIH Publication No. 85–23, revised 1996) and were approved by the University of Wisconsin-Madison Institutional Animal Care and Use Committee (protocol# V005847). Timed-pregnant Sprague-Dawley rats, aged twelve weeks (with a positive plug indicating gestation day (GD) 1), were obtained from Envigo Laboratories (Indianapolis, IN, USA) on GD 3. They were housed in a controlled environment with regulated temperature and a 12:12-h light–dark cycle. The rats were exposed to PFOS at a concentration of 50 μg/mL (CAS #2795-39-3, Sigma Aldrich, St. Louis, MO, USA) through their drinking water from GD 4 to GD 20. As a control group, other rats received drinking water without detectable PFOS. The chosen PFOS dose was based on our previous studies to mimic concentrations found in occupational exposures [85]. The United States Environmental Protection Agency (US EPA, 2016) established a health advisory limit of 70 parts per trillion (ppt) for PFOS in drinking water [91]. The PFOS exposure at 50 µg/mL used in this study is roughly five times higher than this advisory limit, reflecting exposure levels observed in heavily contaminated regions or occupational settings [85,92,93]. Additionally, this concentration is commonly utilized for testing the effects of PFOS on pregnancy and fetal development [43,82,83]. A subset of Control and PFOS-exposed rats were treated with an AT2R agonist, Compound 21 (C21; VicorePharma, Gothenburg, Sweden), at a dose of 0.3 mg/kg/day subcutaneously from GD 15 to 20. The choice of the C21 dosage was informed by previous studies [94,95], which showed its effectiveness in lowering blood pressure and improving complications associated with hypertension, particularly in hypertensive rats, without affecting control rats. Furthermore, Bosnyak et al. (2011) [96] underscored the striking 4000-fold higher selectivity of C21 for AT2R relative to AT1R, highlighting its potential as a candidate for antihypertensive therapy. On GD 20, blood-pressure measurements and uterine artery ultrasounds were performed on the rats. Subsequently, the rats were euthanized using CO_2_ inhalation. Blood samples were taken via cardiac puncture into heparinized vacutainers to obtain plasma. The uterine arteries were collected for vascular reactivity studies and protein isolation. Additionally, the weights of feto-placental units were measured.

### 4.2. Blood Pressure

At GD 20, blood pressures were noninvasively measured using a tail-cuff method (Kent Scientific, Torrington, CT, USA), as previously described [97,98]. Prior to GD 20, rats were acclimated to the restraint warming chamber for 15 min daily for two consecutive days. On the day of blood-pressure measurements, rats were placed in the restraint warming chamber set at 30 °C and allowed to rest for 10 min to promote dilation of peripheral blood vessels and enhance blood flow to the tail. An occlusion cuff and a volume pressure-recording cuff were applied to the base of the tail. The cuff was programmed to inflate and deflate automatically within a 90-s cycle. Blood pressure was recorded and analyzed using Kent Scientific software (Coda 4.2). The first five inflation cycles were used for acclimation, and the subsequent five cycles’ average was considered as the individual mean blood pressure for each rat.

### 4.3. Uterine Artery Ultrasound

On GD19, rats were anesthetized with 2% isoflurane in oxygen and placed on a heated platform for ultrasound imaging. The uterine arteries were examined using a 30-MHz transducer and the Vevo 2100 ultrasound system (Visual Sonics, Toronto, ON, Canada), following established procedures [99]. Briefly, the velocities of the main uterine artery were recorded below the bladder and at the point where the main uterine artery branches from the internal iliac artery. From three consecutive cardiac cycles, peak systolic velocity (PSV), end-diastolic velocity (EDV), the area under the peak velocity–time curve, and the R-R interval was measured. The results from these measurements were averaged. To determine the blood flow velocity distribution, the following formula was used: F = ½ MVπ (D/2)2, where MV represents the mean peak velocity over the cardiac cycle (in cm/s), D stands for the diameter (in cm), and F represents the blood flow (in mL/min). To assess the pulsatility of blood velocity waveforms, the Uterine Artery Resistance Index (RI) was calculated as (PSV-EDV)/PSV, and the Pulsatility Index (PI) was calculated as (PSV-EDV)/MV.

### 4.4. Ex-Vivo Vascular Reactivity Studies

The main uterine artery was carefully excised and freed from any surrounding connective tissues. Arterial ring segments, each measuring 2 mm in length, were then mounted on a wire myograph (Danish Myo Techniques, Aarhus, Denmark) using tungsten wires to enable the recording of isometric tension. Arterial rings were immersed in Krebs physiological solution (KPS) at a temperature of 37 °C and gassed with a 95% O_2_/5% CO_2_ gas mixture, resulting in a pH of 7.4. The KPS consisted of the following components: NaCl, 118 mM; KCl, 4.7 mM; CaCl_2_, 2.5 mM; MgSO_4_, 1.2 mM; KH_2_PO_4_, 1.2 mM; NaHCO_3_, 25 mM; and glucose, 11.1 mM. The rings were allowed to equilibrate in the KPS solution for one hour under resting tension. Subsequently, the arterial rings were normalized using a specialized software package called Myodata (V8.1.13) from Danish Myotechnology. In the case of endothelium-intact arterial rings, extra precautions were taken to prevent any damage to the endothelial layer. For endothelium-denuded arterial rings, the endothelial layer was gently removed by rubbing the interior of the ring with tungsten wire. The successful removal of the endothelial layer was assessed by the absence of relaxation response to acetylcholine (ACh) in arterial rings that were pre-contracted with a submaximal concentration of phenylephrine (PE).

#### 4.4.1. Assessment of Vascular Contractile Responses

The arterial rings were subjected to an 80 mM potassium chloride (KCl) solution until consistent contractions, induced by depolarization, were observed. Following a subsequent round of washing and equilibration with KPS, the vascular contractile responses were assessed by exposing the rings to increasing cumulative doses of PE ranging from 10^–9^ to 3 × 10^–5^ M, as well as Ang II at concentrations ranging from 10^–11^ to 10^–7^ M.

#### 4.4.2. Assessment of Vascular Relaxation Responses

To evaluate endothelium-dependent relaxation, the response to ACh was measured in arteries pre-contracted with PE, using ACh concentrations ranging from 10^–9^ to 10^–5^ M. For assessing endothelium-independent relaxation, the response to sodium nitroprusside (SNP; 10^–9^ to 10^–6^ M) was measured in arteries without endothelium, which was also pre-contracted with PE. The concentration of PE that induced 80% of the maximal response (pEC80) was utilized for precontraction purposes.

### 4.5. Plasma Bradykinin Levels

Plasma bradykinin concentrations were quantified using an enzyme immunoassay kit (Enzo Life Sciences, ADI-900-206, Farmingdale, NY, USA) following the manufacturer’s instructions. The assay’s detection range spanned from 11.7 to 30,000 pg/mL. For each sample, 100 μL of plasma was used in duplicate for the analysis.

### 4.6. Western Blotting

Arteries were homogenized in ice-cold radioimmunoprecipitation assay buffer (Cell Signaling Technology, Danvers, MA, USA) containing a protease inhibitor tablet (Roche, Indianapolis, IN, USA) and phosphatase inhibitor cocktail-2 and -3 (Sigma). Following centrifugation at 14,000× g for 10 min at 4 °C, the supernatant was aliquoted to measure the concentration of protein (Pierce BCA protein assay kit, Thermo Scientific, Waltham, MA, USA). The supernatant was then re-suspended in NuPAGE^®^ sample buffer and reducing agent (Invitrogen; Thermo Scientific, Waltham, MA, USA). A total of 30 μg of proteins, along with Precision Plus Standard (Kaleidoscope, Bio-Rad, Hercules, CA, USA), were loaded into wells on 4% to 12% gradient NuPAGE^®^ Bis-Tris Gels (Invitrogen, Carlsbad, CA, USA). Electrophoresis was conducted at 100 V for 2 h at room temperature, and then the proteins were transferred onto Immobilon-P membranes (Millipore Inc, Billerica, MA, USA) using a Mini Blot Module (Invitrogen) at 20 V for 1 h. The membranes were blocked (5% skim milk) for an hour and then incubated overnight at 4 °C with primary antibodies, including AT1R (rabbit polyclonal, SAB2100073, 1:1000; Sigma, Burlington, MA, USA), AT2R (rabbit monoclonal, ab92445, 1:1000; Abcam, Cambridge, MA, USA), eNOS (rabbit monoclonal, #32027, Cell Signaling Technologies, Danvers, MA, USA), and β-actin (rabbit monoclonal, #4070, 1:5000; Cell Signaling Technologies). After washing, the membranes were treated with secondary antibodies (anti-rabbit conjugated with horseradish peroxidase) for 1 h and detected using Pierce-enhanced chemiluminescence detection kits (Thermo Scientific, Waltham, MA, USA). Densitometric analysis was carried out with Image J software (1.47t). The results were normalized and expressed as ratios of the intensity of a specific band to that of β-actin.

### 4.7. Placental and Fetal Weights

Feto-placental units were extracted from the uterus, and the fetuses were sorted into male and female groups based on their anogenital distance. The corresponding placenta was also separated according to the fetal sex. Any excess fluid was carefully blotted from both the fetuses and placentas. Subsequently, the wet weights of the fetuses and placentas were measured using an electronic scale with an accuracy of ±0.1 mg (Mettler Instrument Corp, Model AE50, Hightstown, NJ, USA).

### 4.8. Statistical Analysis

Statistical analyses were performed using Prism software (GraphPad, version 9, San Diego, CA, USA). The data were presented as the mean ± standard error of the mean (SEM). Two-way analysis of variance tests was conducted, followed by Tukey’s multiple comparisons tests. Cumulative concentration–response curves were analyzed using a four-parameter sigmoidal curve-fitting approach. Contraction responses to PE were expressed as a percentage of its maximal contraction and as percent of 80 mM KCl contraction. Relaxant responses to ACh and SNP were expressed as a percentage of relaxation from the PE-induced contraction. Statistical significance was considered at a *p*-value of less than 0.05.

## Figures and Tables

**Table 1:** Vascular function in Control and PFOS dams with and without C21.

Variable	Control	Control + C21	PFOS	PFOS + C21
Ang II pD_2_	8.73 ± 0.03 ^a^	8.65 ± 0.02 ^a^	9.03 ± 0.05 ^b^	8.75 ± 0.02 ^ac^
Ang II E_max_	111.76 ± 2.73	120.33 ± 5.09	127.14 ± 4.84	121.39 ± 3.44
ACh pD_2_	7.12 ± 0.11 ^a^	7.26 ± 0.06 ^a^	6.58 ± 0.07 ^b^	7.04 ± 0.12 ^ac^
ACh E_max_	98.68 ± 0.73 ^a^	93.50 ± 2.20 ^a^	70.90 ± 4.72 ^b^	85.01 ± 3.72 ^ac^
SNP pD_2_	6.80 ± 0.06	6.82 ± 0.05	6.84 ± 0.07	6.75 ± 0.03
SNP E_max_	97.45 ± 4.13	97.79 ± 4.12	96.45 ± 4.64	97.89 ± 4.19

pD2 (negative log molar concentration that produces 50% effect) is presented as −log [mol/L], and Emax (maximal responses) is presented as percent of maximal contraction or relaxation. All abbreviations are defined in the text. Means with different letters indicate significant differences (*p* ≤ 0.05) among the groups.

**Table 2:** Litter size and fetal sex-ratio in control and PFOS dams with and without C21.

	Control	PFOS	Control + C21	PFOS + C21
Litter size	12.3 ± 2.6	11.9 ± 1.8	13.4 ± 0.37	13.4 ± 0.91
Sex ratio (percent males per litter)	47 ± 3.9%	50 ± 4.5%	48 ± 4.6%	44 ± 4.2%

## Data Availability

All data needed to evaluate the conclusions in the paper are present in the paper. Additional data related to this paper may be requested from the author.
